# Isolated Paramedian Lower Lip Cleft: A Case of a Rare Form of a 28–29 Tessier Cleft

**DOI:** 10.7759/cureus.105138

**Published:** 2026-03-12

**Authors:** Mahmoud Y Aldooma, Hassan S Mohamed Elhadi

**Affiliations:** 1 Oral and Maxillofacial Surgery, Sudan Medical Council, Khartoum, SDN; 2 Dentistry, Sudan Medical Council, Khartoum, SDN

**Keywords:** congenital facial malformation, oral and maxillofacial surgery, paramedian lower lip cleft, rare craniofacial anomaly, tessier cleft 28–29

## Abstract

An isolated paramedian lower lip cleft is an extremely rare congenital anomaly classified within Tessier clefts 28-29. The embryological basis remains incompletely understood, and very few cases have been documented. We report the third isolated case and sixth overall of a paramedian lower lip cleft in a 43-year-old male. Clinical examination revealed a full-thickness cleft of the lower lip on the left side, just lateral to the midline. It presented as a discontinuity of the red line, the vermilion, and the white roll. Surgical repair was performed using a Z-shaped incision on the clefted side and an inverted L-shaped incision on the contralateral side to restore symmetry and function. Postoperative recovery was uneventful, and the patient achieved competent lip function with a satisfactory esthetic outcome. This case highlights a rare variant of Tessier cleft and underscores the importance of documenting such anomalies, expands the clinical spectrum of Tessier clefts 28-29, guides surgical decision-making, builds a more comprehensive understanding, improves outcomes for future patients, and improves awareness among clinicians encountering similar presentations.

## Introduction

Paramedian clefts of the lower lip are very rare. To date, only five cases of paramedian lower lip clefts have been documented, with most cases being syndromic and only two isolated cases. The last case was reported on February 18, 2025, which was considered a typical form of Tessier 28-29 rather than a true cleft [1]. The anatomical classification proposed by Tessier in 1976 is widely accepted as the standard for describing craniofacial clefts [2]. In Tessier's classification, cleft lip and/or mandible midline is classified as number 30, and paramedian lower cleft lip defects are classified as new Tessier 28-29, as proposed by Morritt et al. [3].

Many theories and hypotheses have been proposed to explain the causes of a paramedian lower lip cleft. For example, Warbrick et al. attributed it to a fusion defect between the mandibular prominences of the first branchial arches [4]. Another potential cause is the failure of mesodermal migration and penetrance, in association with the partial or complete failure of growth center differentiation within the developing mandible [5]. Others suggest that a paramedian lower lip cleft is caused by intrauterine trauma (reduction of a multifetal pregnancy) [6], which is associated with craniofacial abnormalities and serious congenital anomalies, including cleft lip (with or without a cleft palate) and facial clefts [7,8]. Although all these cases of paramedian cleft were associated with syndromes or anomalies, only two cases were isolated; the last was reported in 2024 by Dr. Srishti Shailesh Ghorpade [9]. In this case, we report the third isolated and the sixth paramedian cleft of the lower lip.

## Case presentation

Case history

In September 2023, a 43-year-old male presented with a congenital discontinuity of the lower lip, present since birth, with no family history or associated anomalies.

The patient presented to us complaining of a discontinuity of his lower lip on the left side, resulting in disfigurement of his appearance. His parents told him that it had been present since he was born, and they did not seek any treatment for him; no such condition was present in the family. He had no trauma, disease, or surgical procedure to the lower lip. He was not diabetic, hypertensive, allergic to any medications, or under any medications.

Physical examination

**Figure 1:**
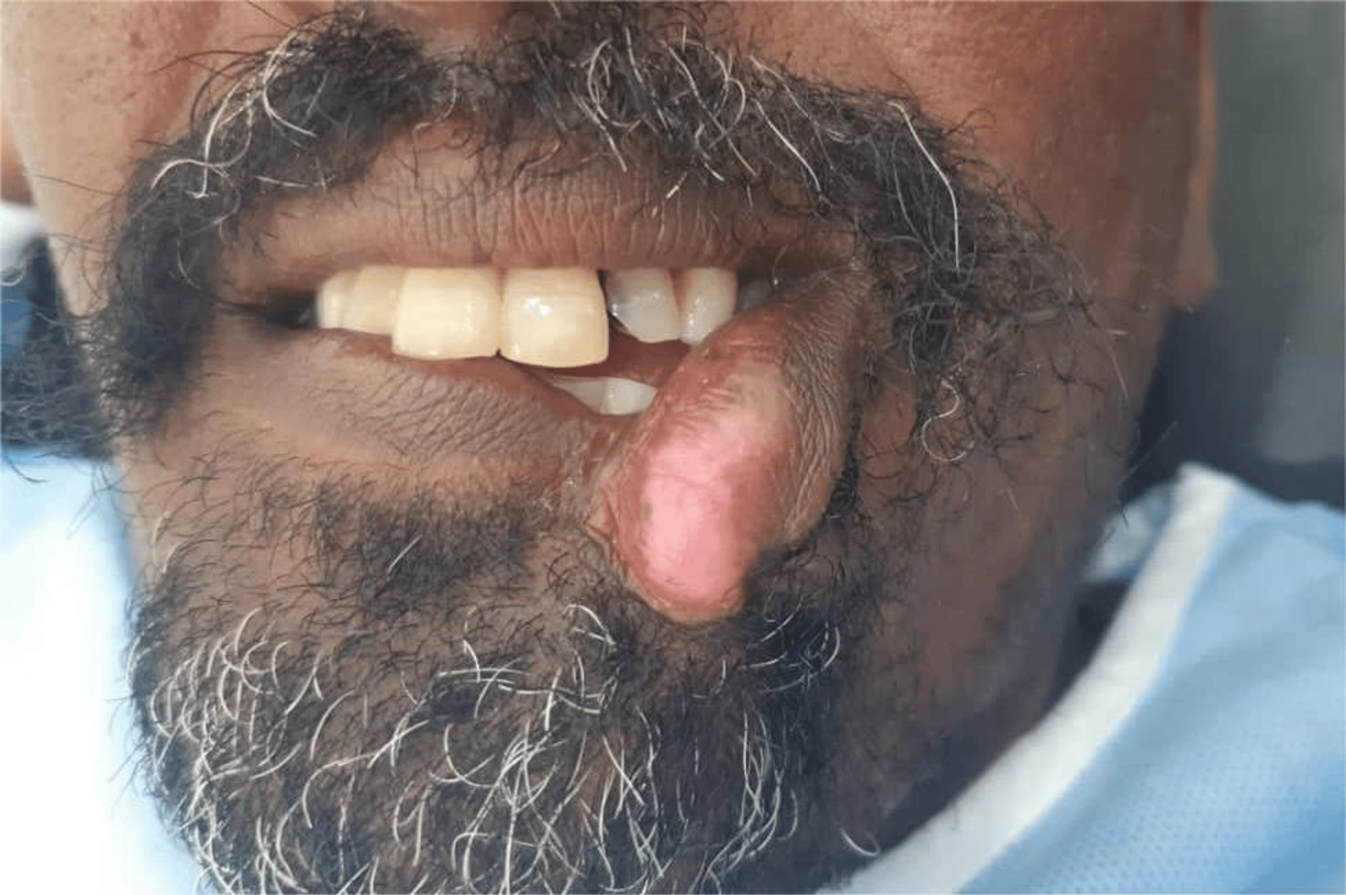
Discontinuity of the red line, vermilion, and the white roll, with the lower lip directed downward and outward Preoperative view showing congenital discontinuity of the paramedian lower lip.

A full-thickness cleft of the lower lip was located on the left side, just lateral to the midline. It presented as a discontinuity of the red line, the vermilion, and the white roll. The lower lip was directed downward and outward on the left, resulting in incompetent lips (Figure 1).

Treatment

**Figure 2:**
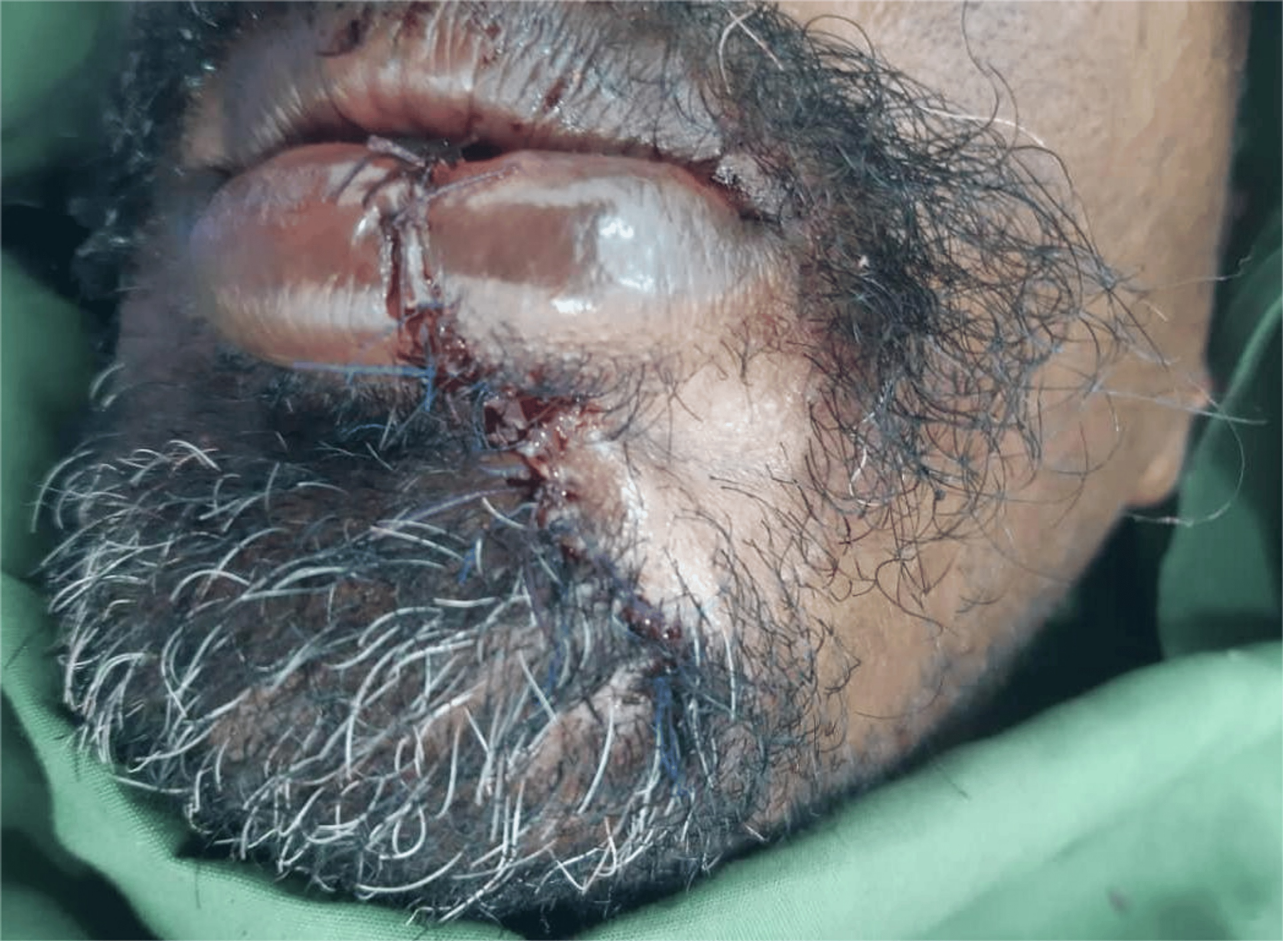
Patient's photo immediately after the operation, with aligned lip vermilion, white roll, and orbicularis oris muscle

**Figure 3:**
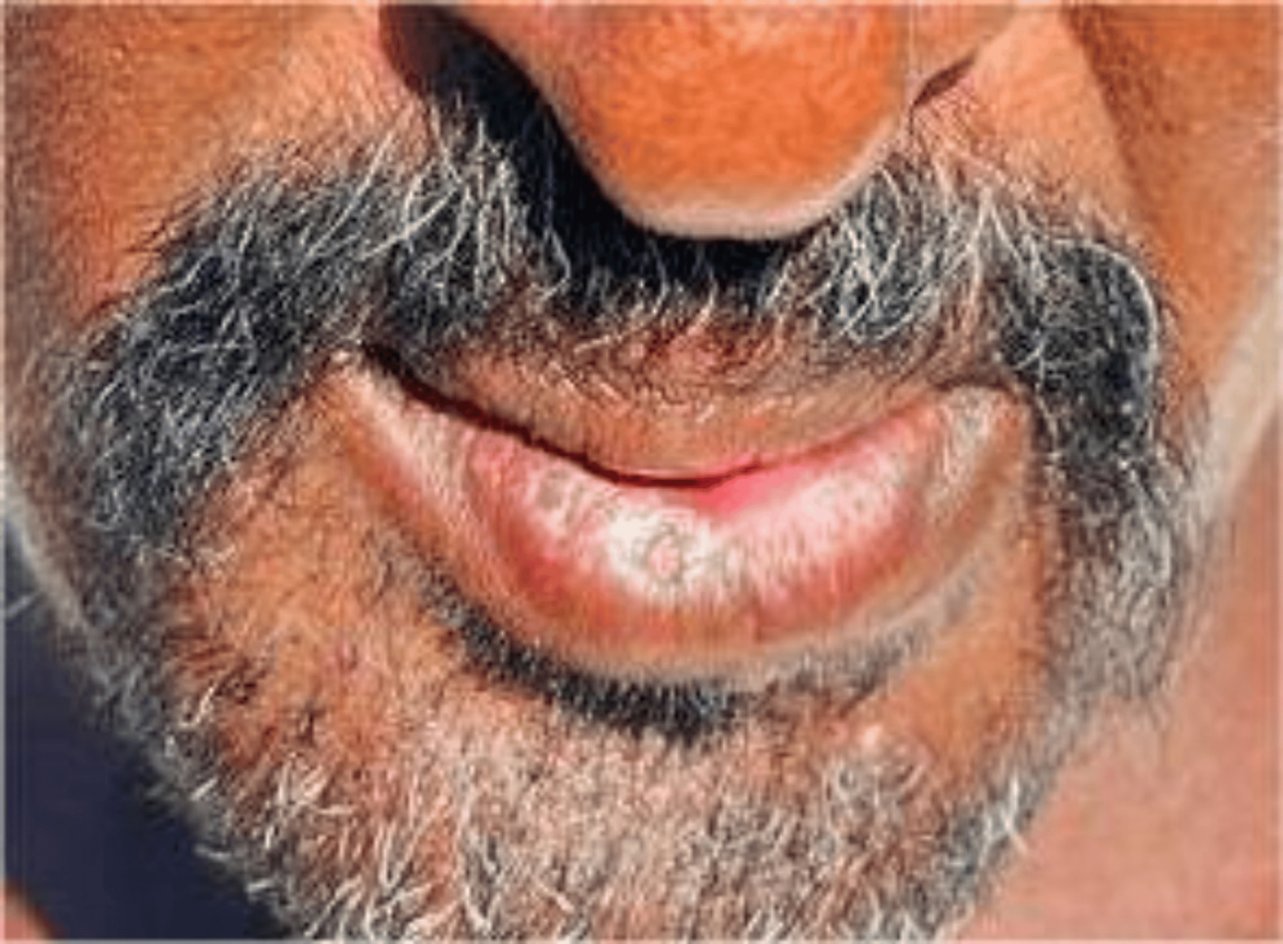
Well-healed scar with competent lips and an acceptable esthetic outcome after two months

The case was operated under local anesthesia. A left inferior alveolar nerve block was performed using lidocaine 2% local anesthesia with epinephrine. Infiltration anesthesia was administered over the left chin area to promote hemostasis. The anesthesia was allowed to take effect for 10 minutes, after which the incision (Z) on the cleft side was made. The incision started at the point where the white roll at the vermilion faded, extended to the skin towards the midline, and then went backward laterally and down to the chin area. A triangular flap of equal distances was made. The incision in the white roll was continued down to the labial sulcus, just before the sulcular depth. On the right non-clefted side, an inverted (L) incision was made, starting at the vermilion, extending downward and outward to the midline, and then downward alongside the cliff for a distance equal to that of the clefted side. The white roll incision was also continued from the vermilion to the sulcular area, and excess skin and mucosa at the cleft area were excised. The abnormal orbicularis oris attachment on the chin area was then detached, and the other non-clefted side was reoriented with it and sutured tightly using size 1 Vicryl. The skin was sutured using 5/0 nylon, starting at critical landmarks (the lower vermilion, the labiomental fold, and the tip of the triangular flap). The lip vermilion, white roll, and orbicularis oris muscle were aligned (Figure 2), and a well-healed scar, with competent lips and an acceptable esthetic outcome, was achieved after two months (Figure 3).

## Discussion

Paramedian clefts are an even rarer entity, with only five cases described in the literature so far [10].

To our knowledge, this case represents only the third isolated occurrence and the sixth overall reported in the literature [2], underscoring both its rarity and clinical significance.

Tessier’s classification provides a framework for characterizing craniofacial clefts, yet isolated lower lip clefts remain underreported and poorly understood [1]. Most documented cases describe paramedian clefts in association with mandibular or alveolar anomalies [3], whereas isolated discontinuity of the lower lip is exceedingly rare.

Literature suggests two main hypotheses: incomplete fusion of mandibular processes versus vascular disruption during lip development [1,4]. Our case, when compared with these two cases, lacks systemic or syndromic features, and this aligns more closely with the incomplete fusion theory.

Many published cases describe lower lip clefts in conjunction with mandibular hypoplasia or commissural defects [1,3]. By contrast, our patient presented with an isolated defect, which highlights the spectrum of presentation, suggesting multiple pathogenic mechanisms.

The challenging point regarding the treatment was that there was no known standardized technique for lower lip cleft treatment when compared to the upper lip cleft case; thus, surgical management has varied considerably. Some authors have advocated rotation or advancement flaps to optimize symmetry and function [2]. By contrast, our approach utilized a Z-shaped incision on the clefted side and an inverted L-shaped incision contralaterally with an extension of the lower limbs in both incisions while keeping them within bear hair.

This technique provided functional competence and esthetic balance, offering improved scar camouflage and contour restoration compared with simpler linear closures described in earlier literature [2]. These differences in surgical approaches highlight the need for individualized management strategies.

Postoperative outcomes in published cases have generally been favorable, with the restoration of oral competence and acceptable cosmetic results [2]. Our findings are consistent with these outcomes, as healing was uneventful and the patient achieved satisfactory functional and esthetic outcomes. Importantly, the tailored use of combined Z and inverted L incisions may represent a refinement of previously described techniques, particularly in achieving bilateral symmetry.

.

## Conclusions

This case illustrates the rare occurrence of an isolated paramedian lower lip cleft without associated anomalies. Early recognition and tailored surgical intervention allowed for a single definitive repair, achieving both functional and aesthetic restoration. The key lesson is that individualized management, guided by the extent of tissue involvement, can yield excellent outcomes even in rare craniofacial anomalies.
